# The effects of diets enriched in omega-3 polyunsaturated fatty acids on systemic vaccinia virus infection

**DOI:** 10.1038/s41598-017-16098-7

**Published:** 2017-11-22

**Authors:** Gwendolyn J. B. Jones, Rachel L. Roper

**Affiliations:** 1 Department of Microbiology and Immunology, Brody School of Medicine, East Carolina University, Greenville, NC, 27834 USA; 2 East Carolina Diabetes and Obesity Institute, Brody School of Medicine, East Carolina University 600 Moye Blvd, Greenville, NC, 27834 United States of America

## Abstract

Omega-3 polyunsaturated fatty acids (PUFA, n-3 fatty acids), the key components of fish and flaxseed oils, are increasingly consumed by the public because of their potential health benefits and are available by prescription for hypertriglyceridemia. However, numerous studies have shown that these compounds are immunoregulatory and immunosuppressive and thus may increase susceptibility to infection. In this study, we tested the effects of the amount of fat and the types of fatty acid in the diet on infection by vaccinia virus, an acute infection that begins in the respiratory tract and spreads by viremia to internal organs. Male C57Bl6 mice (~5 week old) were fed for 3 weeks prior to infection and continuing during infection and recovery one of the following: 1) a normal low fat (13% kcal) diet, 2) a low fat diet containing n-3 PUFAs, 3) a high fat (41% kcal) diet rich in n-3 PUFAs, 4) a high fat n-6 PUFA diet, or 5) a high fat monounsaturated diet. We found no statistically significant differences in the susceptibility of mice to viral infection, morbidity, viral organ titers, recovery time, or mortality with these diets, indicating that, over this approximately 6-week time period, dietary fats did not substantially affect responses to poxviral infection.

## Introduction

Omega 3 or n-3 polyunsaturated fatty acids (PUFAs), the key components of fish and flaxseed oils, are routinely consumed by the general public as food additives or supplements and have emerging clinical utility for the treatment of metabolic, cardiac, inflammatory, and autoimmune diseases^1–5^. n-3 PUFAs are available as prescription supplements (Lovaza/Omacor, Vascepa, Epanova, Omtryg) for treating hypertriglyceridemia^6,7^, and several studies now support the use of n-3 PUFAs in the prevention and treatment of Alzheimer’s disease^8–10^. However, many studies report that dietary n-3 PUFAs are immunosuppressive and may increase morbidity or mortality to infection^3,11–16^, and transgenic Fat-1 mice that have increased endogenous production of n-3 PUFAs (the fat-1 gene catalyzes conversion of n-6 PUFAs into n-3 PUFAs) are more susceptible to pulmonary tuberculosis^12^. n-3 PUFAs have been reported to alter lipid rafts, inhibit Toll like receptor clustering and NF-kB activation, and suppress antigen presentation to CD4 + T cells, formation of the immunologic synapse, cytokine secretion, and T helper cell activation^3,5,17–19^. A number of studies have been performed on the effects of dietary n-3 PUFAs on bacterial pathogenic infections, including: *Mycobacterium tuberculosis*, *Salmonella typhimurium*, *Streptococcus pneumoniae*, *Pseudomonas aeruginosa*, *Escherichia coli*, *Staphylococcus aureus*, *Citrobacter rodentium*, *Helicobacter hepaticus* and *pylori*, and, *Listeria monocytogenes*, (reviewed in^3^). While reports suggest that N-3 PUFAs may have beneficial effects against extracellular pathogens, there is more evidence that they may be harmful during infection with intracellular pathogens, especially at higher doses^3^. Mice fed a diet enriched in n-3 PUFAs for 2 weeks have been reported to have increased viral load and mortality when infected with influenza virus^11^, but very few studies have been performed on systemic viral infections in mammals.

The objective of this study was to assess the effects of increased dietary n-3 PUFA levels on viral load, survival, and recovery after infection with the poxvirus vaccinia virus, a model in which virus initially infects the respiratory tract and then spreads systemically by viremia to infect internal organs^20–22^. Recovery requires a complex innate and adaptive immune response with prominent B and T lymphocyte functions^20–23^. We assessed viral load in lung and liver, liver being especially affected by dietary fat intake. The studies were conducted comparing a low fat (LF) purified control mouse normal diet (ND, 13% kcal fat made with soybean oil as the fat source), a LF n-3 PUFA diet (13% kcal fat with flaxseed and fish oils), and a high fat (HF, 41% kcal) n-3 PUFA diet to compare the amount of n-3 PUFAs. In addition, we assessed the effects of a HF n-6 PUFA diet (vegetable, safflower, and coconut oils) to explore the specificity of the type of PUFA, as well as a HF monounsaturated (MUFA, olive oil) diet to compare poly- vs. mono- unsaturated dietary fat^24,25^. Mice were fed diets for 3 weeks prior to infection with vaccinia virus and continued on their respective diets while monitoring morbidity, mortality, viral load, and recovery over 3 additional weeks (total 6 weeks). This study shows that while n-3 PUFAs can be immunosuppressive, increases in dietary n-3 PUFAs for several weeks do not necessarily increase disease associated with systemic viral infection.

## Results

### Effects of dietary fats on viral morbidity, mortality, and recovery

**Figure 1:**
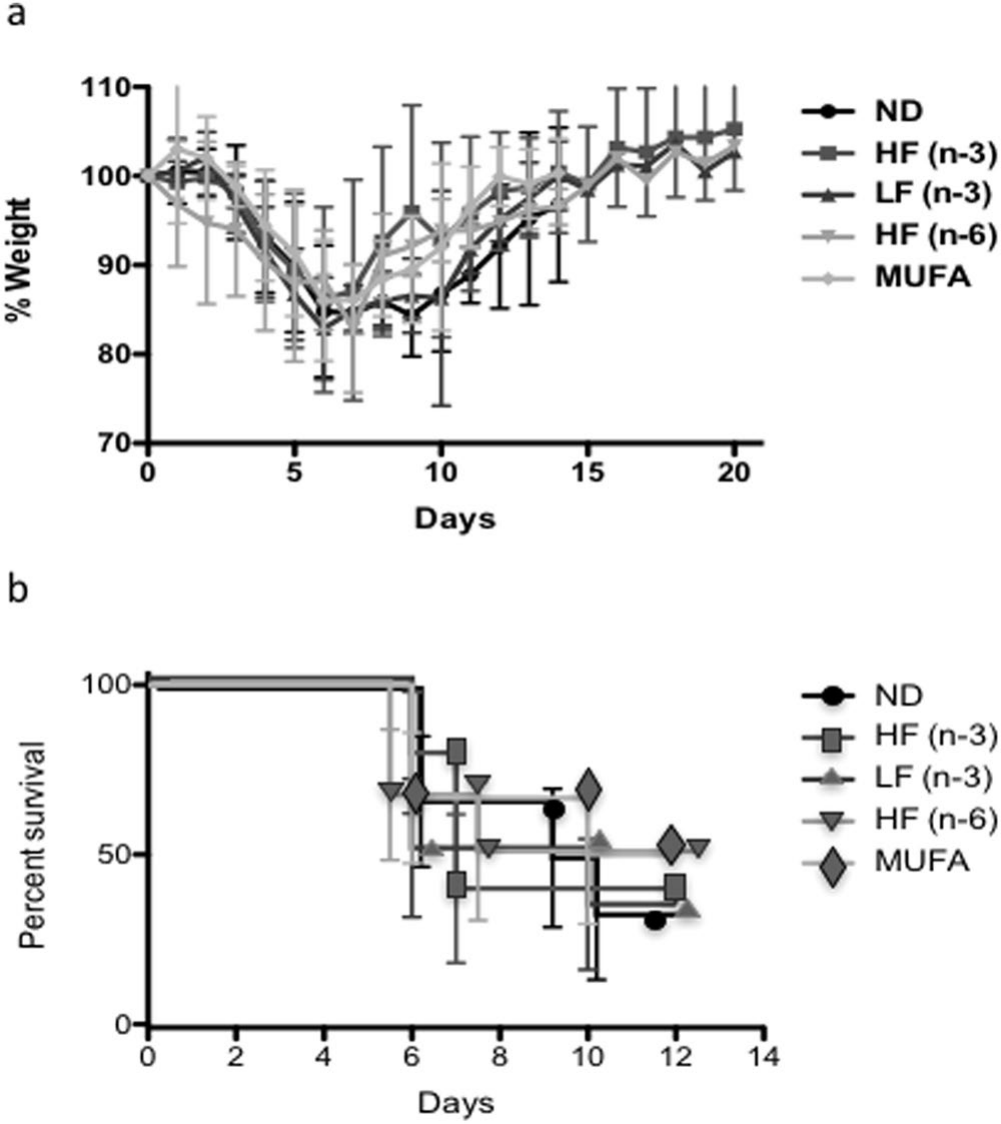
Effects of dietary fats on morbidity, survival and recovery in response to infection with vaccinia virus. (*a*) Weight loss and recovery of mice fed for 3 weeks with: a control Normal Diet (ND), High fat n-3 PUFA (HF n-3), Low fat n-3 PUFA (LF n-3), High fat n-6 PUFA (HF n-6), or High fat monounsaturated (MUFA). Mice were intranasally infected with 5 × 10^5^ pfu vaccinia virus and maintained on the same diets throughout the experiment (6 weeks total). (**b**) Survival curves; mice were sacrificed if they lost 20% body weight. Data are means ± SEM, n = 7–10 mice per diet. Symbols were enlarged and overlaid manually (1b) in order to improve readability of the figure.

To test the hypothesis that the HF n-3 PUFA diet would lower survival in response to viral disease, we measured effects of the 5 diets in Table 1 with varying amounts and types of fats: a low fat (13% kcal) Normal Diet (ND), high fat (41% kcal) polyunsaturated PUFA n-3 (HF n-3), low fat PUFA n-3 (LF n-3), high fat PUFA n-6 (HF n-6), and high fat monounsaturated (MUFA). Mice were fed diets for 3 weeks, and then infected by the respiratory route with virulent vaccinia virus and continued on their respective diets for 20 additional days. There were no statistically significant differences in morbidity, measured as weight loss, between groups (Fig. 1a). Mice in all groups lost similar amounts of body weight to day 6, when several mice lost more than 20% of their body mass and were sacrificed. Mice from each group died between day 6 and 10 (Fig. 1b), but no differences between diet groups were significant. All mice surviving to day 10 recovered, and surviving mice were monitored for recovery as weight gain. Recovery rates were similar for all groups to day 20 post-infection (Fig. 1a) when mice had recovered to their original body weights. In this experiment, the fat composition of the diets did not significantly affect the rate or amount of weight loss, survival, or recovery. The HF n-3 PUFA group did not demonstrate an overall higher fatality rate than in mice fed ND.

**Table 1:** Composition of diets.

Ingredients	ND	LF n3 PUFA	HF n3 PUFA	HF n6 PUFA	HF MUFA
% fat	5	5	20	20	20
Kcal fat	13%	13%	41%	41%	41%
Flaxseed	0	23.1	92.5	0	0
Fish	0	23.1	92.5	0	0
Soybean	50.0	3.8	15.0	30.0	15.0
Safflower	0	0	0	125.0	0
Coconut	0	0	0	45.0	0
Olive	0	0	0	0	185.0
Casein	220.0	185.0	220.0	220.0	220.0
L-Cystine	2.5	2.5	3.0	3.0	3.0
Corn starch	370.0	370.0	173.9	173.9	173.9
Maltodextrin	140.0	140.0	140.0	140.0	140.0
Sucrose	150.0	150.0	150.0	150.0	150.0
Cellulose (fiber)	50.0	50.0	50.0	50.0	50.0
Mineral mix, AIN-93M	35.0	35.0	42.0	42.0	42.0
Vitamin mix, AIN-93	15.0	15.0	18.0	18.0	18.0
Choline bitartrate	2.5	2.5	3.0	3.0	3.0
TBHQ	0.02	0.02	0.06	0.06	0.06
Vegetable Shortening	0.0	0.0	0.0	185.0	0.0

HF - high fat, LF - low fat, MUFA - monounsaturated fatty acid, ND - normal diet, PUFA - polyunsaturated fatty acid.

### HF and LF PUFA diet effects on survival in response to challenge by vaccinia virus

**Figure 2:**
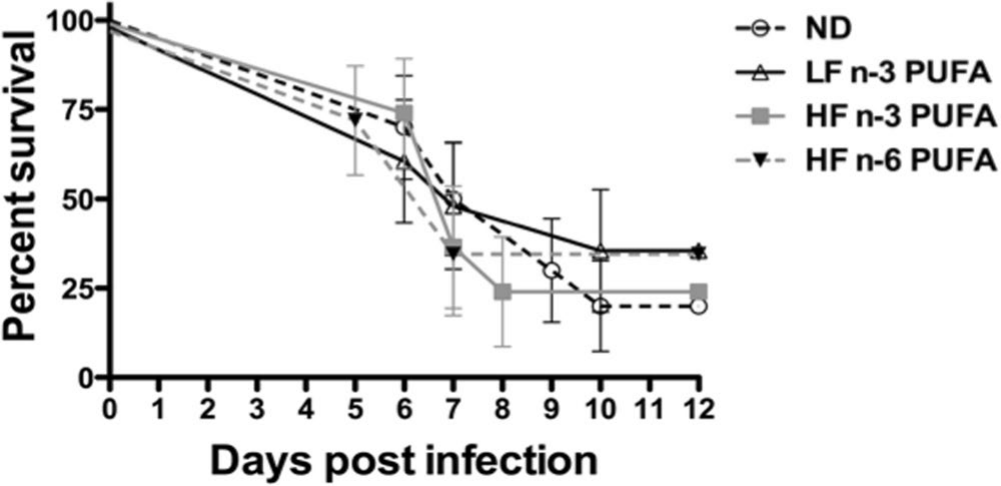
Effects of PUFA-enriched diets on survival of vaccinia virus challenge. Mice were fed for 3 weeks with: a control Normal Diet (ND), High fat n-3 PUFA (HF n-3), Low fat n-3 PUFA (LF n-3), or High fat n-6 PUFA (HF n-6). Mice were intranasally infected with 10^6^ pfu vaccinia virus and maintained on the same diets throughout the experiment. Mice were sacrificed if they lost 20% body weight. Data are means ± SEM, n = 7–10 mice per diet. Differences were not statistically significant.

To further study the specific effects of the n-3 and n-6 PUFA enriched diets, we repeated these experiments with a higher dose of vaccinia virus (10^6^ pfu/mouse) to give a stronger viral challenge to see if differences could be uncovered. In these experiments, most mice again died between day 6 and 10 (Fig. 2). 75% of the HF n-3 PUFA mice were dead by day 8, while ND mice took until 10 days to reach equivalent survival rates. However, the differences between groups were not statistically significant. The median survival for the ND was 8 days. The HF n-6 and HF n-3 PUFA diet fed mice showed a median survival of 7 days, and the LF n-3 PUFA had a median survival of 8.5 days. Overall survival rates did not significantly differ between the diets.

### Virus Organ Titers

**Figure 3:**
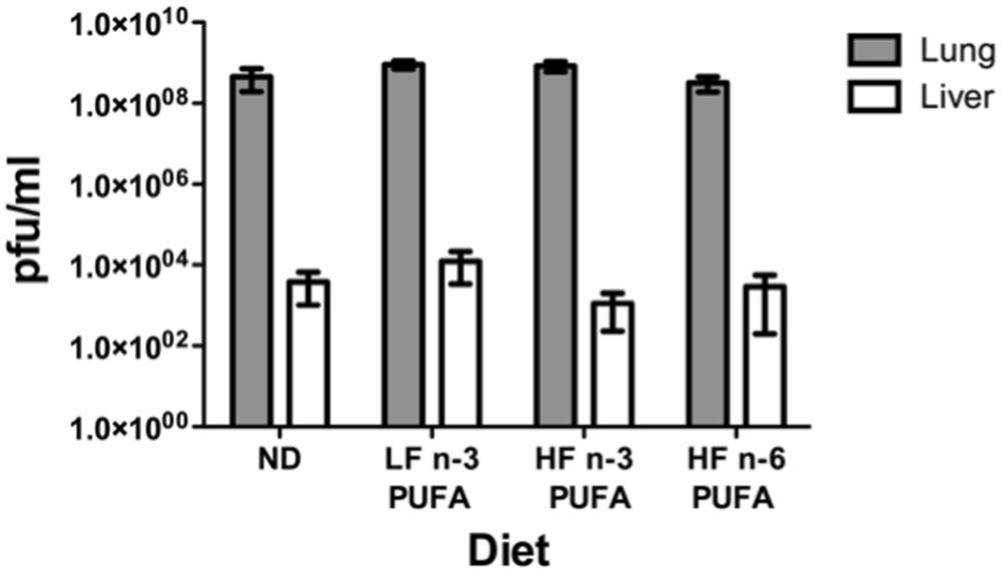
Effects of PUFA-enriched diets on viral titers. Amounts of infectious virus were measured in lung and liver 6-7 days post vaccinia virus infection for mice fed: a control Normal Diet (ND), High fat n-3 PUFA (HF n-3), Low fat n-3 PUFA (LF n-3), or High fat n-6 PUFA (HF n-6) diets for 3 weeks. Data are means ± SEM, n = 7–10 mice per diet, except HF n-6 PUFA, which is n = 4.

It had been shown previously that these HF diets suppressed activity, increased body weight, and lowered the respiratory exchange ratio; and the HF n-6 PUFA, LF and HF n-3 PUFA diets reduced serum triglycerides and lowered the size of the lipid droplets in the liver relative to ND^24,25^. Since different fat diets have been shown to have immune effects as well as changes in liver, we further addressed if there were differences in the amount of infectious virus (titers) replicating in liver in mice infected by the respiratory route by a virus that also spreads to infect the liver. It was possible that virus might replicate better in liver, even if our measurements on weight and mortality were not sensitive enough to detect these changes. Therefore, mice were administered a respiratory viral challenge, and lung (primary site of infection) and liver titers were measured on mice sacrificed on days 6-7 post infection, the time period of expected peak viral load as shown previously^20,22^. On average, lung and liver titers were between 10^8^ −10^9^ and 10^3^ −10^4^ pfu/mL respectively (Fig. 3). There were no significant differences between liver and lung titers in mouse groups fed these different diets despite differences in serum and liver triglycerides.

## Discussion

Our data show that, despite reported negative effects of consumption of increased quantities of dietary fats (particularly n-3 PUFAs) on immune responses and susceptibility to pathogens^3,11,26^, short term feeding (3–6 weeks) of such diets does not necessarily increase disease or interfere with recovery from acute, systemic, viral infection. A high fat diet rich in n-3 PUFAs did not significantly increase morbidity or mortality in response to infection with the poxvirus vaccinia virus. In our diet groups, morbidity (measured as weight loss), mortality, and recovery followed similar patterns. Both the HF n-3 PUFA and HF n-6 PUFA fed mice lost weight and died faster by one day compared to normal diet (day 7 compared to day 8), but differences were not statistically significant. The amount of virus in relevant organs also was not increased by increased dietary consumption of PUFAs (n-3 or n-6). Together these results suggest that this level of dietary n-3 PUFA is not enough to significantly impair functioning of the immune system required to protect mice from this virulent virus challenge, as survival and recovery rates were similar in all groups. Virulence studies with vaccinia virus typically include 5 mice per group^20–22^, but we extended studies up to 7–10 mice to ascertain whether a significant difference could be measured. However, no differences were evident, even in a strong challenge in which 75% of the mice died.

Changes in dietary fats have pleotropic effects in the body, and the biochemical mechanism of action on inflammation and immunity is not fully understood. Many proposed mechanisms have been described, including effects on lipid rafts, receptor clustering, and multiple intracellular signaling pathways^3^. It was reported previously that this HF n-3 PUFA diet (mixed fish/flaxseed oil) dose reduced mouse activities and lowered the respiratory exchange ratio^25^. Feeding of the HF n-3 PUFA diet described here significantly changed the lipid composition (including the n-6/n3 ratio) of splenocytes (mostly lymphocytes and accessory cells) in only one day, and differences were substantially increased after feeding for 17 days^24^. However, these measurable metabolic and cellular effects did not translate directly into differences in resilience to viral infections. Perhaps effects would have been greater with increased feeding times, but most studies reporting effects described experiments in which diets were fed from 2–5 weeks^3^, similar to our study of feeding for 3 weeks prior to infection and then continuing on the diets during infection for an additional 3 weeks (6 weeks total).

Prior studies on the effects of increased n-3 PUFA have shown mixed results in bacterial infection models, with some increases or decreases in various measures of morbidity, inflammation, pathogen load and mortality [reviewed in 3]. n-3 PUFAs may have direct effects on the pathogen, on pathogen interactions with cells, on inflammation, or on the innate and adaptive immune responses; so differences in reported results may be due to the specific pathogen or type of disease progression in the body^3^. In some cases, excess inflammation may be the cause of disease, while in others, inflammation may be crucial for recovery. In addition, differences may be due to the type of immune response required for eradication of infection. Extracellular pathogens require a different type of response for clearance than intracellular pathogens, including viruses, that require specific T lymphocyte activity to target and kill infected cells. Previous reports suggested that increases in dietary n-3 PUFA may increase morbidity and mortality from intracellular pathogens such as influenza virus^3,11^, but our data show that this is not true for all viral infections. Vaccinia virus infecting by the respiratory route and then spreading systemically to organs including liver, which is known to be particularly affected by dietary fats, showed little difference in morbidity or mortality with variations in dietary fats. This should be reassuring for those employing increased dietary n-3 PUFAs therapeutically or preventatively, although much more research is needed, particularly on their effects on viral infections, as very few viral infection systems have been studied^3,11^. In addition, effects in mice or other mammals may not translate directly into humans because of metabolic and immune response differences. Further studies will be needed in humans to fully understand the effects of dietary PUFAs on human responses to and recovery from infection.

Many dietary studies evaluating the efficacy of n-3 PUFAs in differing model systems have relied on low doses of fish or flaxseed oils. With animals, investigators have commonly used ~5% (wt/wt) fish or flaxseed oil as intervention, which corresponds to approximately 2–6% of total energy as n-3 PUFA (depending on the source of the n-3 PUFA^27^. This dose, especially of fish oil, is often selected to model the intake of n-3 PUFA in specific populations (e.g. Greenland Eskimos, Japanese fishing villages) that consume n-3 PUFAs in the range of 1–6% of the total energy and display low incidence of metabolic and/or inflammatory diseases^28–30^. Some studies have tested the effects of fish or flaxseed oils at higher doses with mixed results on metabolic and immune parameters^3^. High doses of n-3 PUFAs could have a unique therapeutic niche for treating specific metabolic and/or inflammatory diseases, or could exert negative side effects, raising potential safety issues. We show here that a diet enriched in either n-3 or n-6 PUFAs (even to 20% fat, 41% kcal) did not significantly lower survival to vaccinia virus infection, at least with short-term (~6 week) feeding in mice.

## Methods

### Mice and diets

All mice were housed in the East Carolina University Department of Comparative Medicine animal facility and kept in conventional conditions with full access to food and water throughout the study. All procedures were approved by the East Carolina University Animal Care and Use Committee (K157) and in accordance with recommendations for proper care and use of laboratory animals. Male C57BL/6 mice (~4–6 weeks old) were placed for 3 weeks on one of four experimental diets. The mice were administered two low fat (LF), 5% fat by weight, and three high fat (HF), 20% kcal fat by weight, diets^18,23^. All diets were purified and are designated as follows: Normal Diet (ND), High Fat polyunsaturated n-3 (HF n-3), Low fat polyunsaturated n-3 (LF n-3), High fat polyunsaturated n-6 (HF n-6), and high fat monounsaturated (MUFA), Table 1 and previously published^18,23^.

For the ND and LF n-3 PUFA diets, ~13% of the total kcal is from fat. For the HF diets, ~41% of the total kcal is from fat. For the HF n-3 PUFA diet, of the 41% of the total kcal from fat, ~10-11% was from α-linolenic acid and ~4-5% was from eicosapentaenoic (EPA) and docosahexaenoic (DHA) acids. The LF n-3 PUFA diet was designed such that the fatty acid profiles between the LF and HF n-3 PUFA diets are identical. The ND has nearly the same amount of linoleic acid (~7%) as the HF n-3 PUFA diet. Therefore, the HF n-3 PUFA diet is matched with the LF n-3 PUFA diet in terms of the fatty acid profile and with the ND control in terms of the amount of linoleic acid. Diets were routinely analyzed for their fatty acid composition by gas chromatography as previously described^18^.

### Viral infection

All viral infections and procedures were carried out in Animal Biosafety Level 2 + facilities by vaccinated personnel. After 3 weeks of specific diet feeding, groups of 7–10 C57/Bl6 mice per diet were anesthetized with isoflurane and infected intranasally with 5 × 10^5^ (Fig. 1) or 10^6^ (Fig. 2) plaque forming units (pfu) per mouse of purified vaccinia virus strain Western Reserve in 18 ul of 10 mM Tris, 150 mM NaCl, 1 mM EDTA, pH 7.5 as previously described^20–22^. Viral titers were measured on solutions used to infect the mice on the day of infection to confirm virus dose. The mice were maintained on their diets (~6 weeks total), weighed daily, and sacrificed if they lost 20% or more of their original body weight (as required by the Animal Care and Use Committee). All mice were euthanized via isoflurane overdose followed by cervical dislocation to confirm death.

### Viral organ titers

All viral infections and procedures were carried out in Biosafety Level 2 facilities by vaccinated personnel. Viral titers were determined as previously described^20,22^. Groups of C57/Bl6 mice (n = 7–10 mice per diet) were sacrificed at days 6-7 post-infection (when they had lost 20% of their original body weight) and the lung and livers were placed into 1 mL of cold RPMI medium. The organs were then freeze-thawed three times, homogenized, and sonicated. Viral replication was measured by titrating the organ suspension on BS-C-1 monolayers followed by staining 40 h later with 0.1% crystal violet in ethanol. Plaques were counted independently by two investigators (M.H, G.J) and confirmed by a third person (R.R.)

### Data and statistical analyses

The data are from several independent experiments. Each independent experiment consisted of one mouse per diet and was replicated at least seven times. Therefore, an n of 7 indicates one mouse per diet repeated seven separate times for reproducibility. Data were analyzed using GraphPad Prism. Survival curves in response to infection were analyzed using a log-rank Mantel-Cox test. *P* values less than 0.05 were considered statistically significant. Symbols were enlarged and overlaid on the graph manually in order to improve readability of Fig. 1b.

### Data availability statement

All data will be made available upon request.
